# RNA–Protein Dynamics of the Segmentation Clock and Its Implications in Developmental Disorders

**DOI:** 10.3390/ijms27209199

**Published:** 2026-10-16

**Authors:** Willem B. Jay Tan, Ami Kobayashi, Kuniyasu Niizuma

**Affiliations:** 1 Department of Neurosurgical Engineering, Graduate School of Biomedical Engineering, Tohoku University, Sendai 980-8575, Japan; willemtan88@gmail.com (W.B.J.T.); kuniyasu.niizuma.c3@tohoku.ac.jp (K.N.); 2 Division of Applied Biomedical Science and Biotechnology, School of Health Sciences, IMU University, Kuala Lumpur 57000, Malaysia; 3 Department of Neurology, Brigham and Women’s Hospital, Harvard Medical School, Boston, MA 02115, USA; 4 Department of Medical Science and Innovation, Institute of Medical Research, Tohoku University, Sendai 980-8574, Japan; 5 Department of Translational Neuroscience, Tohoku University Graduate School of Medicine, Sendai 980-8575, Japan; 6 Department of Neurosurgery, Tohoku University Graduate School of Medicine, Sendai 980-8574, Japan

**Keywords:** RNA processing, RNA modification, epitranscriptomics, segmentation clock, somitogenesis, post-transcriptional regulation, mRNA decay, translational control

## Abstract

Somitogenesis is orchestrated by the segmentation clock, a highly conserved developmental oscillator that generates periodic gene expression waves to establish precise somite boundaries along the vertebrate body axis. While transcriptional feedback circuits and signaling gradients are recognized as central determinants of clock dynamics, accumulating evidence indicates that RNA-centered regulation provides an important layer of temporal control. We therefore focus primarily on established RNA-regulatory processes, including nascent RNA synthesis, pre-mRNA processing, transcript stability and degradation, and translational control, and examine how these processes interact with protein turnover and intercellular signaling to shape oscillation period, amplitude, and synchronization. Because the molecular implementation of the clock differs among vertebrates, we distinguish species-specific systems throughout: mouse studies primarily define hairy and enhancer of split 1 (Hes) 7/lunatic fringe (Lfng)/Delta-like canonical Notch ligand (Dll) 1 dynamics, zebrafish studies define Hes-related bHLH transcriptional repressor (her) 1/her7/deltaC/deltaD dynamics, chick studies established cyclic c-hairy1/lfng expression, and human HES7/LFNG dynamics are examined mainly in stem-cell-derived presomitic mesoderm (PSM) models. These mechanisms are used to formulate experimentally testable hypotheses about phase-dependent RNA stability, translation, and developmental state transitions. Together, the segmentation clock provides a quantitative framework for distinguishing established RNA–protein mechanisms from emerging RNA-regulatory hypotheses and for understanding how their disruption may contribute to congenital vertebral disorders.

## 1. Introduction

The segmented organization of the vertebrate body axis represents one of the most remarkable examples of precision in embryonic pattern formation and has fascinated developmental biologists for centuries. In the seventeenth century, Marcello Malpighi described repetitive structures along the trunk of chick embryos, observations that would later be recognized as somites, the transient embryonic units that give rise to the vertebral column, skeletal muscle, tendons, and dermis [1,2,3]. Somites are generated through the progressive segmentation of the presomitic mesoderm (PSM), a dynamic embryonic tissue that undergoes periodic partitioning into bilaterally symmetric epithelial blocks through a process known as somitogenesis [3]. The extraordinary temporal accuracy of this process is governed by the segmentation clock, an evolutionarily conserved molecular oscillator that produces rhythmic waves of gene expression across the developing PSM and determines the timing of somite formation [4].

The concept of a developmental oscillator was first established through the discovery of cyclic c-hairy1 expression in chick embryos, providing the first direct link between periodic gene expression and the regular production of somites [5]. Subsequent studies identified related but species-specific oscillatory systems, including Hes7 and Lfng in mouse, her1/her7 and deltaC/deltaD in zebrafish, and HES7/LFNG oscillations in human stem-cell-derived presomitic mesoderm models [6,7,8,9,10,11,12]. These components participate in evolutionarily conserved regulatory themes involving Notch, Wnt, fibroblast growth factor (FGF), and retinoic acid signaling, but their gene repertoires, paralog usage, coupling mechanisms, and experimental evidence differ among organisms. The pace of the segmentation clock also varies markedly across vertebrates, ranging from approximately 30 min in zebrafish to approximately 2–3 h in mouse and 4–6 h in human in vitro models (Table 1) [11,13,14].

Historically, the segmentation clock has been understood primarily through transcriptional feedback loops and extracellular signaling networks. Emerging evidence indicates that RNA metabolism contributes actively to oscillator dynamics, but the relevant transcripts must be interpreted in species context. For example, rapid turnover of mouse Hes7/Lfng transcripts and zebrafish her1/her7 transcripts contributes to the short feedback delays required for oscillatory behavior in their respective systems [43,44,45,46,47,48]. Live imaging of zebrafish her1 further demonstrates that stochastic transcriptional bursting is subsequently shaped by RNA decay, translation, and tissue-level coupling [21]. These findings support a general role for RNA kinetics in developmental oscillators while avoiding the assumption that mouse Hes7/Lfng and zebrafish her1/her7 constitute interchangeable molecular circuits.

The significance of RNA regulation extends beyond developmental mechanisms and provides a critical framework for understanding human congenital disease. A growing number of genetic disorders are now recognized as disorders of RNA biology, arising from defects in pre-mRNA splicing, transcript stability, translation, localization, or RNA modification [43,44,45,46]. Congenital vertebral malformations and segmentation defects of the vertebrae (SDV), including spondylocostal dysostosis (SCD), congenital scoliosis (CS), and Klippel–Feil syndrome (KFS), provide compelling examples of how disruption of segmentation clock components can compromise vertebral patterning and lead to lifelong anatomical abnormalities [49,50,51]. Importantly, disease-associated mechanisms are not always explained by alterations in DNA sequence alone; rather, they frequently reflect defects in the molecular processes that determine how RNA transcripts are generated, processed, interpreted, and eliminated [52,53,54,55]. In parallel, emerging evidence suggests that epitranscriptomic modifications, including N6-methyladenosine (m6A), pseudouridylation, and RNA editing, can regulate transcript stability, translation efficiency, and cell-state transitions during development [56,57]. However, whether and how these RNA modifications contribute to segmentation clock dynamics remains largely unexplored, representing a major unanswered question.

Recent technological breakthroughs provide unprecedented opportunities to address these questions. Live RNA imaging, single-cell and spatial transcriptomics, and human stem-cell-derived models of somitogenesis, including gastruloids, segmentoids, and somitoids, enable the segmentation clock to be interrogated with previously unattainable temporal and spatial resolution [12,17,58]. These approaches have revealed that oscillatory gene expression arises from the integrated activity of transcriptional circuits, RNA processing pathways, translational regulation, and cell–cell communication.

Importantly, the strength of evidence differs across these regulatory layers. In this review, we use the term RNA dynamics in a deliberately restricted sense to refer to the temporal control of transcript synthesis, processing, stability, degradation, and translation. Protein turnover, metabolism, and signaling are discussed only where they alter the timing, amplitude, robustness, or functional interpretation of these RNA-derived oscillatory signals. This distinction allows the review to retain an RNA-centered focus while recognizing that clock output is generated by an integrated RNA–protein kinetic system. Established RNA-processing and transcript-level mechanisms therefore form the core of the analysis, whereas protein-, signaling-, and metabolic-level mechanisms are treated as complementary modulators and are evaluated according to the evidence hierarchy described in subsection “*Evidence Hierarchy and Policy for Preprints*” in Section 2. Methodology.

## 2. Methodology

This article is a narrative review rather than a systematic review or meta-analysis. A structured literature search was performed in PubMed, Scopus, Web of Science, and Google Scholar, and was updated through 18 September 2026. Search terms included combinations of “segmentation clock”, “somitogenesis”, “presomitic mesoderm”, “Hes7”, “Lfng”, “her1”, “her7”, “Delta/Notch”, “RNA processing”, “mRNA decay”, “translation”, “ribosome”, “metabolism”, “RNA modification”, “epitranscriptomics”, “spondylocostal dysostosis”, and “vertebral segmentation defects”. Because terminology and gene nomenclature differ among vertebrate species, searches were supplemented with species-specific gene names and related terms where appropriate. Reference lists of key primary studies and relevant reviews were also screened to identify additional mechanistic studies that were not captured in the initial database searches.

Primary research addressing molecular mechanisms of the segmentation clock, somitogenesis, axial development, or human vertebral segmentation disorders was prioritized. Studies were included when they provided experimental evidence relevant to segmentation clock dynamics, somitogenesis, presomitic mesoderm or axial development, RNA and protein kinetics, intercellular signaling, timing, boundary formation, clock termination, or human vertebral segmentation disorders. Conference abstracts, student manuscripts, and studies without a clear relationship to somitogenesis or the mechanistic questions addressed in this review were excluded. When multiple studies supported the same mechanistic point, peer-reviewed primary studies with direct experimental evidence were preferentially cited, with additional reports included when they provided distinct species-specific, methodological, clinical, or mechanistic information.

Selected studies were synthesized according to the stage of the segmentation process and, importantly, according to the strength and location of evidence. Studies were excluded when they were conference abstracts, student manuscripts, non-English articles without an accessible English translation, or publications without a clear relationship to segmentation clock biology, somitogenesis, axial development, or the specific RNA/protein mechanisms considered in this review. Studies from unrelated tissues or disease systems were included only when they provided a clearly defined mechanistic principle relevant to an unresolved question in segmentation clock biology; such evidence was explicitly identified as indirect rather than being treated as direct support for a clock mechanism. The selected literature was synthesized according to both the stage of the segmentation process and the strength of the evidence. Accordingly, the review distinguishes direct measurements of segmentation clock dynamics from evidence obtained during somitogenesis or axial development without direct clock measurements, from mechanistic inference based on other tissues or biological systems, and from hypotheses requiring experimental validation. Human genetic associations are interpreted separately from experimentally measured clock phenotypes, and computational or modeling results are not treated as direct mechanistic evidence unless they are experimentally validated. This evidence hierarchy is used throughout the text, figures, and tables to avoid assigning equivalent certainty to observations of different evidentiary strength.

### Evidence Hierarchy and Policy for Preprints

For interpretive consistency, evidence is classified into four levels: Level I, directly demonstrated in the segmentation clock, with experimental measurement or perturbation of oscillation period, amplitude, phase, synchronization, propagation, or clock-to-boundary conversion; Level II, demonstrated in somitogenesis or axial development but without direct measurement of clock oscillations; Level III, inferred from another tissue, developmental context, or biological system and therefore mechanistically suggestive but not demonstrated in the PSM clock; and Level IV, hypothetical, computationally predicted without direct experimental validation, or proposed as a future testable mechanism. Experimentally constrained mathematical models are discussed together with the level of the underlying empirical evidence, rather than being used to upgrade evidentiary certainty.

Preprints were included only when they addressed a recent and directly relevant mechanistic question for which no equivalent peer-reviewed publication was available at the time of the final search. Preprints are explicitly identified as such in the text, evidence table, and reference list, and their conclusions are described as provisional. Representative mechanisms and their corresponding levels of evidence are summarized in Table 2.

## 3. Initiating the Clock: Transcriptional Onset and RNA Processing

### 3.1. Emergence of Oscillatory Transcription and Nascent RNA Control

The establishment of the segmentation clock occurs within the broader context of early embryonic axis formation, during which precise regulation of lineage specification and transcriptional programs progressively establishes the PSM. Following fertilization, the human embryo undergoes successive mitotic divisions to form the blastocyst, which subsequently escapes the zona pellucida and initiates implantation at Carnegie stage 4 (CS4) [70]. By CS6, gastrulation begins with the emergence of the three primary germ layers, ectoderm, mesoderm, and endoderm, followed by the formation of the primitive streak (PS) at the posterior epiblast between CS7 and CS9 [71,72]. Comparable developmental transitions occur in mouse embryos between Theiler stages (TS) 6–8 [73]. These early patterning events establish the axial progenitor populations that ultimately generate the paraxial mesoderm and initiate the segmentation program. A comprehensive chronological overview of these developmental transitions has been described by Zhai et al., providing an essential framework for understanding how embryonic patterning progresses towards somitogenesis [71].

PSM formation requires appropriate specification of neuromesodermal and mesodermal progenitors before oscillatory gene expression can emerge. ZEB1 and ZEB2 have been implicated in this developmental context, but they are not established components of the segmentation clock oscillator itself. Studies in human stem-cell-derived mesodermal precursors and somitoid/gastruloid models indicate that perturbing ZEB-family activity can alter mesodermal identity, axial elongation, and paraxial mesoderm formation [58,74,75]. We therefore retain ZEB proteins only as brief developmental context for establishing the cellular substrate in which the clock operates, rather than as part of the RNA-regulatory core of the review.

Following PSM specification, spatially restricted transcriptional programs define the molecular landscape in which the segmentation clock operates. In the developing human embryonic tail, spatial transcriptomic analysis identifies distinct anterior and posterior domains, including posterior TBXT/SOX2-positive axial progenitor states and organized anterior–posterior distributions of signaling-associated transcripts [76]. By contrast, mechanistic studies of Msgn1/Tbx6 regulation and somite maturation cited here are derived primarily from mouse models [77,78]. A recent livestock pluripotent-stem-cell embryo-model study is available only as a preprint [79] and is therefore treated as provisional comparative developmental evidence rather than as direct evidence for the human or mouse segmentation clock. Wnt and retinoic-acid-associated spatial programs are discussed only where the cited species and experimental context are specified. This separation prevents observations from distinct vertebrate systems from being combined into a single evidentiary claim.

Beyond transcriptional activation, precise control of transcript persistence and degradation is equally critical for maintaining developmental programs. Failure to appropriately eliminate key PSM transcripts can result in severe patterning abnormalities. Persistent overexpression of *tbx16* leads to impaired craniofacial and ocular development, whereas sustained *msgn1* expression disrupts posterior body formation and prevents proper notochord development in zebrafish [80]. These findings illustrate that developmental timing relies not only on the activation of lineage-specific transcripts but also on their controlled clearance.

These early mesoderm-patterning data establish the developmental context in which the segmentation clock emerges, but they do not by themselves establish a human disease mechanism. To avoid conflating developmental expression with clinical causality, human genetic evidence is considered separately in Section 8.

### 3.2. Establishing RNA Oscillation and Transcript Turnover

Following establishment of the PSM, the molecular machinery of the segmentation clock is initiated through precisely coordinated waves of oscillatory transcription. Among the earliest clock-associated transcripts, Lfng serves as a critical molecular marker of the transition from an undifferentiated PSM state into a self-organizing oscillatory system. In mouse embryos, an initial two-fold pulse of Lfng expression is detected before the onset of short periodic oscillations between Theiler stages (TS) 7 and TS8, coinciding with the initiation of somitogenesis [18]. Lfng encodes Lunatic fringe, a beta1-3 N-acetylglucosaminyltransferase that modifies components of the Notch pathway. Importantly, Fringe effects are context dependent and should not be summarized as a universal reduction in Notch responsiveness to Delta-like ligands. In the mouse PSM, Okubo et al. showed that Lfng contributes to clock synchronization through a trans-repression mechanism in which Lfng in a signal-sending cell modulates Dll1 function and thereby reduces Notch activity in a neighboring cell [19]. More recent work further indicates that Lfng and Dll3 can cooperate through cis interactions to modulate Dll1/Notch1 signal-sending activity [81]. Thus, Lfng is best considered a context-dependent modulator of Notch coupling rather than a simple inhibitor of Delta-induced receptor activation.

Interestingly, the earliest waves of Lfng transcription appear to arise independently of canonical Notch signaling in mouse embryos. A previous report examined mouse Lfng dynamics and showed that initial spatiotemporal waves persisted after pharmacological inhibition of gamma-secretase or genetic disruption of canonical Notch signaling; early Lfng oscillations also persisted in Hes7-deficient mouse embryos [18]. These findings support a sequential model in which oscillator initiation can precede full engagement of canonical Notch-mediated coupling. In this framework, the onset of oscillatory transcription is distinguished from the later role of Notch signaling in intercellular coupling, phase alignment, stabilization, and synchronization of neighboring oscillators in the mouse PSM [17,18,19]. This mouse result should not be interpreted as evidence for zebrafish lfng oscillations; Reference [18] instead compared the mouse findings with earlier zebrafish studies of other clock components. Zebrafish notch-dependent coupling is therefore discussed separately in Section 4.2 using her1/her7 and deltaC/deltaD data. Whether an analogous temporal sequence occurs in human PSM development remains to be established.

The transition from early Lfng activity to Notch-coupled oscillation illustrates how pathway timing is progressively stabilized during segmentation. The developmental roles of individual Notch-pathway components are discussed here at the mechanistic level; human variant interpretation and gene–disease relationships are considered separately in Section 8.

**Figure 1:**
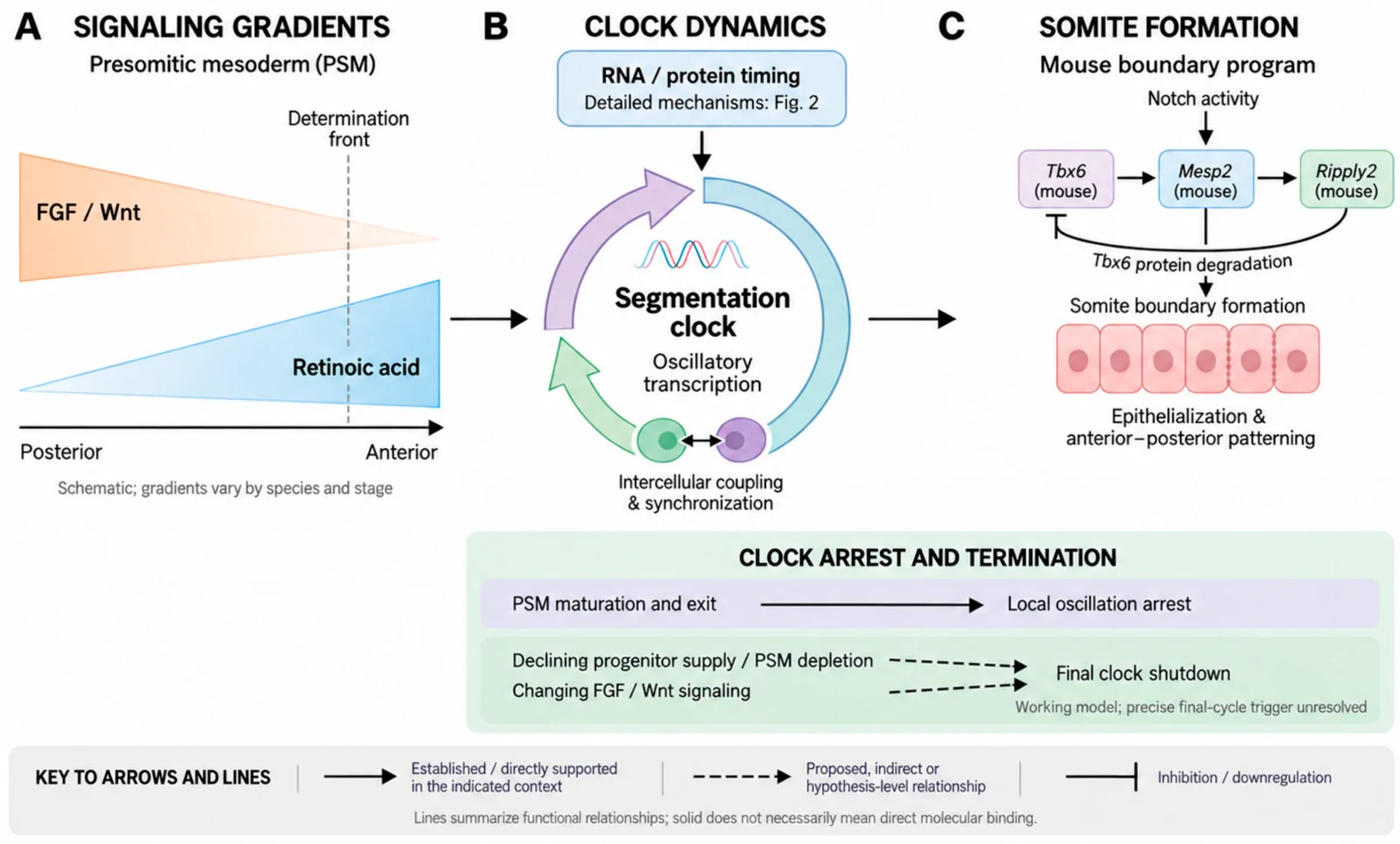
From segmentation clock dynamics to somite patterning. Schematic overview of how RNA regulation integrates signaling gradients, oscillatory gene expression, tissue patterning, and disease during vertebrate somitogenesis. (**A**) Signaling gradients: Schematic distributions of posterior-high fibroblast growth factor (FGF)/Wnt signaling and anterior-high retinoic acid provide positional context along the presomitic mesoderm (PSM). Their interaction with cellular maturation contributes to the spatial organization of somitogenesis. The gray dotted vertical line marks the determination front and represents a positional landmark rather than an uncertain regulatory relationship. Gradient profiles and their functional contributions vary among species and developmental stages. (**B**) Signaling gradients: Schematic distributions of posterior-high fibroblast growth factor (FGF)/Wnt signaling and anterior-high retinoic acid provide positional context along the presomitic mesoderm (PSM). Their interaction with cellular maturation contributes to the spatial organization of somitogenesis. The gray dotted vertical line marks the determination front and represents a positional landmark rather than an uncertain regulatory relationship. Gradient profiles and their functional contributions vary among species and developmental stages. (**C**) Clock dynamics: Oscillatory transcription within PSM cells, together with intercellular coupling and synchronization, generates coordinated segmentation clock activity. RNA and protein kinetics contribute to the timing and robustness of these dynamics. The detailed RNA/protein regulatory processes and a mouse Notch-coupling example are presented separately in Figure 2 to avoid duplication. Somite formation: A mouse boundary-regulation module illustrates how transient developmental signals contribute to stable segmental patterning. Tbx6 and Notch activity promote *Mesp2* expression; Mesp2 subsequently induces *Ripply2*, whose protein product promotes Tbx6 protein degradation. This regulatory relationship restricts the Tbx6-dependent transcriptional program and contributes to the positioning and refinement of segmental boundaries. The molecular program is followed by somite boundary formation, epithelialization, and anterior–posterior compartmentalization. The depicted mouse module should not be interpreted as an identical regulatory sequence in all vertebrate species. The lower panel distinguishes local oscillation arrest during PSM maturation and exit from the final cessation of segmentation along the developing axis. Declining posterior progenitor supply, PSM depletion, and changing FGF/Wnt signaling are shown as contributors to a working model of final clock shutdown. Dashed connections indicate that their precise causal sequence and the mechanism determining the final oscillatory cycle remain unresolved. Created in BioRender. Table, W. (2026) https://BioRender.com/rmov6qq.

As PSM cells transition from progenitor states towards segmentation competence, oscillatory transcription becomes integrated with opposing signaling gradients that provide positional information along the anterior–posterior axis. During this transition in mice, tailbud-derived progenitors expressing *Tbx6* and *Hes7* experience a progressive reduction in posterior FGF/Wnt activity, including decreased expression of *Fgf8*, *Fgf17*, and *Wnt3a*, as cells acquire PSM identity [17]. This generates a posterior-to-anterior gradient of FGF/Wnt signaling, counterbalanced by an opposing retinoic acid (RA) gradient, which together define the position of the determination front where oscillatory information is translated into stable somite boundaries (Figure 1) [17].

**Figure 2:**
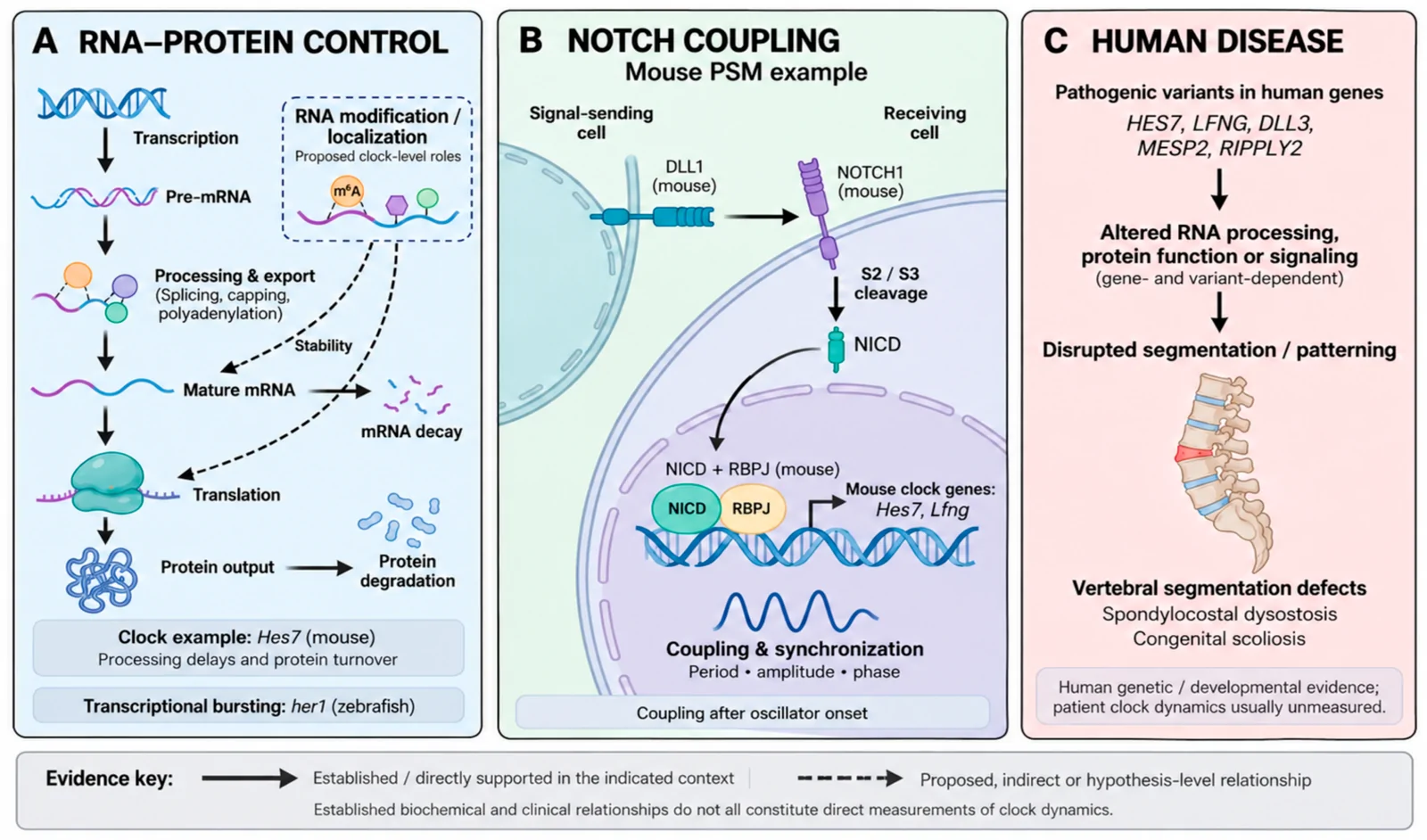
RNA–protein control, Notch coupling, and human disease-associated disruption of the segmentation clock. (**A**) RNA–protein control: The main pathway depicts transcription, pre-mRNA processing and nuclear export, mature mRNA, translation, and protein production. Processing includes splicing, capping, and polyadenylation. Separate branches indicate mRNA decay and protein degradation, distinguishing protein production from subsequent turnover. Mouse *Hes7* provides an experimentally supported clock-specific example of the importance of transcript-processing delays and protein turnover. Transcriptional bursting of zebrafish *her1* is presented as a separate, species-qualified observation rather than incorporated into the mouse signaling circuit. Dashed arrows from the RNA-modification/localization inset indicate proposed effects on transcript stability and translation within the segmentation clock. In this inset, localization refers to additional regulatory spatial targeting or sequestration, distinct from the established nuclear-export step in the main pathway. The schematic does not imply that every general RNA-processing step has independently demonstrated effects on clock period. (**B**) Notch-mediated coupling in mouse PSM: DLL1 on a signal-sending cell activates NOTCH1 on an adjacent receiving cell. Sequential S2 and S3 cleavage releases the Notch intracellular domain (NICD), which enters the nucleus and participates in an RBPJ-containing transcriptional complex regulating clock-associated genes, including mouse *Hes7* and *Lfng*. The panel emphasizes the contribution of Notch signaling to intercellular coupling, phase coordination, and sustained tissue-level synchrony after oscillator onset; it does not present canonical Notch signaling as a universal requirement for initiating the earliest oscillatory transcription. DLL3 is not depicted as a canonical trans-activating ligand. Uppercase DLL1, NOTCH1, and RBPJ labels denote mouse proteins, whereas italicized *Hes7* and *Lfng* denote mouse genes. (**C**) Human disease associations relevant to segmentation clock dysfunction: Pathogenic variants in human *HES7*, *LFNG*, *DLL3*, *MESP2*, and *RIPPLY2* are associated with congenital vertebral segmentation disorders. Depending on the gene and variant, molecular consequences may involve altered RNA processing, protein function, or signaling, contributing to disrupted segmentation and vertebral patterning. The illustrated outcomes include vertebral segmentation defects, spondylocostal dysostosis, and congenital scoliosis. Human genetic and developmental evidence is distinguished from direct measurement of oscillator behavior: patient clock dynamics are generally unmeasured, and the depicted disorders should not all be classified as RNA-level defects. Created in BioRender. Tan, W. (2026) https://BioRender.com/rmov6qq.

RNA expression patterns are central to establishing and maintaining these signaling landscapes. For example, loss of *lrp6a*, a wnt pathway co-receptor, results in skeletal abnormalities in goldfish, demonstrating the requirement for intact wnt-mediated transcriptional programs during axial development [40]. Similarly, in mice, dynamic expression of *Cdh2*, a downstream target of *Hes7*, forms a posterior-to-anterior mRNA gradient within the PSM and contributes to the regulation of cell adhesion and tissue organization during segmentation [82]. The robustness of these spatial gradients is further reinforced by extracellular matrix architecture, heterogeneous receptor distribution, and ligand retention mechanisms within the developing embryo [83]. Together, these findings illustrate that segmentation requires not only oscillatory RNA production but also precise spatial control of transcript distribution and signaling responsiveness.

Within this molecular environment, the core negative-feedback architecture is implemented by different genes in different vertebrates. In mouse PSM, Hes7 is a principal oscillatory bHLH transcriptional repressor and Lfng provides a cyclic Notch-pathway output, whereas zebrafish use the related her1 and her7 repressors within a distinct paralogous network [84,85,86,87,88]. In zebrafish, her1 homodimers and her1/her7-containing repressor complexes regulate her gene transcription, and notch-dependent coupling contributes to maintenance of coherent tissue-level oscillations [87,88].

The timing of these oscillations is critically determined by RNA kinetics. Rapid transcript production, processing, and degradation are essential because even small alterations in RNA half-life can alter the oscillation period and segmentation fidelity. In mice, *Hes1* expression is highly dependent on Notch signaling, as pharmacological inhibition with DAPT rapidly suppresses *Hes1* transcription within hours, whereas disruption of FGF/Wnt signaling has limited direct effects on transcript production [89]. However, FGF signaling remains essential for maintaining the determination front, as its inhibition accelerates differentiation and produces abnormal segmentation phenotypes, including shortened tails and asymmetric somite formation [17,89].

In contrast, zebrafish segmentation provides an additional example of how RNA oscillation is coupled to signaling dynamics through post-transcriptional regulation. The expression of *her1* is tightly associated with oscillations in extracellular signal-regulated kinase (ERK) activity, which occurs in an opposing phase as cells progress from posterior PSM towards differentiation [90,91]. ERK activity is required for normal somitogenesis, as prolonged inhibition disrupts axis extension and prevents boundary formation [90]. Mechanistically, her1 protein regulates ERK dynamics through recruitment and stabilization of dual-specificity phosphatase 4 (dusp4), promoting dephosphorylation of activated ERK (ppERK) [91]. Notably, although HER1 stabilizes DUSP4 protein, *dusp4* mRNA itself does not oscillate with *her1*, revealing an additional layer of regulation occurring beyond transcriptional control. This finding illustrates that segmentation clock dynamics emerge from coordinated regulation at multiple molecular levels, including RNA production, protein stability, and signaling feedback.

Collectively, establishment of the segmentation clock can be viewed as a progressive maturation of oscillatory control, but the molecular implementation is species specific. In mouse embryos, early Lfng activity can precede full canonical Notch coupling, after which Dll1/Notch signaling contributes to synchronization of Hes7/Lfng dynamics [18,19]. In zebrafish, her1/her7 oscillations and deltaC/deltaD-mediated coupling provide a distinct experimental framework for phase coordination [24,25,26,27,28,29,30,31,32,33,87,88]. FGF/ERK and Wnt signaling provide spatial information in both systems, although their downstream gene networks and experimental readouts differ.

Importantly, the integrity of this system relies on precise transcript regulation. Premature degradation or altered stability of key transcripts can disrupt clock periodicity and segmentation. For example, accelerated decay of Cdh2 mRNA reduces FGF-associated signaling and prematurely terminates Hes7 oscillations [82]. This example supports transcript turnover as a determinant of developmental timing without implying that all segmentation disorders are primarily RNA-mediated; the clinically established mechanisms are distinguished in Section 8.

## 4. Maintaining Periodicity and Synchronization Through RNA Dynamics

The maintenance of segmentation clock oscillations requires not only the generation of rhythmic gene expression but also precise coordination between temporal oscillations and spatial progression along the anterior–posterior (AP) axis. The classical “clock and wavefront” model has provided a foundational framework for understanding somitogenesis, proposing that oscillatory gene expression programs function as an intracellular clock, while a maturation front or “wavefront” determines the position at which cells become competent to form somites [2]. In this model, the interaction between temporal oscillations and positional information supplied by opposing signaling gradients establishes the periodic pattern of somite formation [92].

However, recent zebrafish studies have challenged the necessity of a distinct wavefront mechanism and proposed an alternative ‘timer’ model in which oscillatory PSM cells progressively change their dynamics as they approach differentiation [22,93]. Rohde et al. demonstrated in zebrafish that posterior PSM cells exhibit sustained her1 oscillations, whereas anterior PSM cells display fewer oscillatory cycles and earlier arrest of expression [22]. These observations suggest that zebrafish cells initiate a developmental countdown upon exiting the tailbud, with the duration of this timer influencing the number of her1 cycles experienced before differentiation and boundary formation. This zebrafish model is discussed as a species-specific mechanistic proposal rather than being assumed to apply identically to mouse or human PSM.

Importantly, this molecular timer is not independent of extracellular signaling but is actively regulated by signaling gradients that influence RNA dynamics. FGF signaling acts as a key modulator of oscillatory duration, with increased FGF activity extending the number of oscillatory cycles and altering somite size [94]. Conversely, attenuation of FGF signaling accelerates differentiation and disrupts segmentation, demonstrating that FGF activity is required for maintaining oscillatory competence while its progressive decline along the PSM establishes the appropriate timing window for differentiation [89]. Thus, rather than functioning as a binary positional signal, the FGF gradient appears to regulate the kinetics of the segmentation clock by modulating the molecular processes that determine oscillator persistence.

The relationship between oscillation arrest and differentiation further suggests that segmentation timing is governed by multiple interconnected regulatory mechanisms. In zebrafish, Rohde et al. observed that cessation of her1 oscillations and cellular differentiation are not completely coupled, raising the possibility that parallel molecular timers or distinct sensors interpret overlapping temporal information [22]. Whether equivalent timer architectures operate in mouse, chick, or human PSM remains unresolved and should be tested directly in those systems.

RNA dynamics provide a compelling mechanistic framework for addressing this question. The period of an oscillatory gene expression cycle is determined not only by transcriptional feedback but also by the cumulative delays introduced by RNA synthesis, pre-mRNA processing, nuclear export, translation, and protein degradation. Small alterations in any of these processes can significantly influence oscillator period, synchrony, and robustness. For example, delayed transcript maturation or inefficient RNA turnover can extend the duration of negative-feedback loops, whereas accelerated RNA degradation may prematurely terminate oscillations.

Although the identity of the proposed timer remains unresolved, previous studies have suggested that it may involve transcriptional regulators, microRNA cascades, or RNA-processing mechanisms [22]. In particular, the kinetics of pre-mRNA splicing, transcript maturation, translation efficiency, and nuclear transport represent attractive candidates because they directly influence the delay between transcriptional activation and functional protein production. Such molecular delays are increasingly recognized as fundamental determinants of biological oscillators across diverse systems and may represent a critical component of segmentation clock periodicity.

### 4.1. Maintaining the Clock: Turnover, Translation and Metabolic Coupling

Once oscillatory programs are established within PSM, their timing and robustness are also shaped by regulatory processes that act downstream of RNA production. This subsection therefore considers protein degradation, translation, and metabolic state as complementary kinetic layers rather than RNA-regulatory mechanisms themselves. They are included only where experimental perturbation changes clock period, wave propagation, synchronization, or developmental output, thereby showing how RNA-derived oscillatory information is converted into and constrained by functional protein and signaling states.

A key example of this principle is the regulation of Notch intracellular domain (NICD) stability, which determines the duration and strength of Notch signaling during somitogenesis. As previously discussed, *LFNG* oscillations become dependent on Notch activity after the initiation of segmentation, indicating that precise control of Notch pathway dynamics is essential for maintaining clock function [17]. Recent studies using human induced pluripotent stem cell (hiPSC)-derived PSM models have demonstrated that disruption of NICD degradation profoundly alters segmentation clock dynamics [59,60]. Targeted inhibition of F-box and WD repeat domain-containing protein 7 (FBXW7), an E3 ubiquitin ligase responsible for ubiquitin–proteasome system (UPS)-mediated NICD degradation, or mutation of the conserved S2513 residue within the NOTCH1 PEST domain prolonged NICD stability and increased intracellular NICD accumulation [59,60].

FBXW7-mediated degradation acts as a critical buffering mechanism that restricts Notch activity within an appropriate temporal window. Although FBXW7 is not an RNA-regulatory factor, this example is relevant to the central theme because the lifetime of NICD constrains how oscillatory RNA output is translated into signaling duration and tissue-level dynamics. Increased NICD stability elevates several Notch-responsive genes, including NRARP, HES1, and LFNG, but does not simply strengthen the clock; instead, abnormal propagation of HES7 waves is observed in hiPSC-derived PSM cells [59]. Thus, protein turnover provides a downstream kinetic constraint on RNA-generated oscillations. We therefore treat FBXW7–NICD regulation as a complementary protein-level mechanism, not as evidence of direct RNA regulation. These complementary RNA- and protein-level mechanisms, together with Noth-mediated coupling and disease-associated disruption, are summarized schematically in Figure 2.

In addition to proteostasis, peer-reviewed experimental evidence indicates that segmentation clock activity is coupled to cellular metabolism. In mouse PSM explants, increasing glycolytic flux through supplementation with fructose-1,6-bisphosphate (FBP) or its analogue 2,5-anhydro-D-mannitol (2,5-AM) slowed segmentation clock oscillations and produced longer somites, whereas reduced glycolytic activity accelerated oscillatory dynamics [61]. Because these experiments directly measured clock behavior following metabolic perturbation, they are classified as Level I evidence. Other metabolic mechanisms are considered separately according to their experimental and publication status.

Enhancer-mediated gene regulatory network (eGRN) is a map displaying how the relatively distant enhancer segments within a strand of DNA act on turning a particular gene on or off. This analysis has further revealed that FBP-responsive transcriptional changes were associated with regulons controlled by Transcription Factor 7-like 2 (Tcf7l2), a downstream effector of canonical Wnt signaling [61]. TCF7L2 expression is detected within the anterior PSM during developmental stages corresponding to active somitogenesis, suggesting a potential role in integrating metabolic status with axial patterning [95,96]. Given the established role of TCF7L2 in regulating glucose metabolism and metabolic adaptation [97,98], these findings suggest a reciprocal relationship between metabolic state and developmental signaling, whereby changes in glycolytic activity influence transcriptional programs that subsequently regulate oscillator behavior.

A recent preprint provides additional Level I-type experimental observations linking oxygen homeostasis to mouse segmentation clock dynamics. In a conditional Hif1a-loss model in the mouse PSM, abnormal Hes7 and Lfng expression accompanied disrupted oscillations and vertebral defects, and hyperoxic rescue restored clock behavior [62]. HIF1α loss is associated with altered Hes7 and Lfng expression and segmentation clock abnormalities, supporting an indirect link between oxygen-sensitive regulation and clock-associated transcription [62]. Because this work remains a preprint, we treat the mechanistic conclusion as provisional despite the direct measurement of clock phenotypes. Evidence that transcription factors can bind RNA in other systems [99] is therefore classified separately as Level III inference rather than part of the demonstrated mouse mechanism.

A recent mouse preprint used long-term live imaging to report that Hes7 oscillations persist throughout the tailbud and PSM during ongoing cell proliferation and rapidly resume following mitosis [63]. The same study used perturbation and entrainment approaches to connect cell-cycle progression with clock dynamics and somite scaling. These observations are classified as direct (Level I) with respect to the measured clock phenotype, but are explicitly treated as provisional because the study has not yet undergone conventional journal peer review.

The same preprint also reports that cell division occurs throughout the mouse PSM rather than being restricted to posterior regions, suggesting that both tailbud and PSM proliferation contribute to tissue expansion [63]. Related peer-reviewed observations in avian systems support contributions from coordinated cell movement and tissue expansion [100,101,102]. Because tissue-growth observations do not by themselves demonstrate oscillator regulation, they are interpreted as Level II evidence unless directly coupled to measured clock dynamics.

### 4.2. Synchronizing Oscillations Through Species-Specific Notch Ligand Dynamics

The generation of regularly spaced somites requires more than autonomous oscillations within individual PSM cells; it also depends on the ability of neighboring oscillators to maintain a shared phase relationship. Without intercellular coupling, stochastic differences in transcriptional timing between individual cells would progressively accumulate, leading to phase drift, desynchronization, and defective segment formation. Notch signaling is a central mediator of this coupling mechanism, but its role in synchronization must be distinguished from oscillator initiation. In this review, “clock initiation” refers to the emergence of oscillatory transcriptional activity, whereas “Notch-dependent synchronization” refers to the subsequent intercellular coupling, phase coordination, and stabilization of oscillations across the tissue. The earliest oscillatory transcription can arise before canonical Notch coupling is fully engaged, whereas subsequent Notch-dependent communication promotes coherent tissue-level oscillations [18,19,24,25,26]. Figure 2 therefore depicts Notch as a coupling and synchronization pathway after oscillator onset, not as the universal trigger of the first oscillatory cycle.

In the mouse PSM, DLL1 is a canonical trans-activating Notch ligand with direct evidence for a role in intercellular coupling. Chimeric analyses showed that Dll1-mediated signaling is required for normal synchronization, while Lfng can modulate Dll1 function in signal-sending cells to repress Notch activity in neighboring cells [19]. This trans-repression model emphasizes that LFNG does not simply reduce receptor responsiveness in all contexts; rather, it can alter the behavior of the signal-sending cell and thereby tune coupling strength and timing. Accordingly, DLL1 is depicted as the canonical trans ligand in Figure 2.

DLL3 is biologically distinct from DLL1. Mammalian DLL3 is an atypical Delta-like protein that is largely retained in intracellular compartments and primarily acts through cis/intracellular inhibitory or modulatory interactions rather than functioning as a conventional trans-activating ligand. In the segmentation clock, DLL3 and LFNG genetically and biochemically interact, and LFNG-dependent modification of DLL3 can influence DLL1/NOTCH1 signal-sending activity through cis-regulatory mechanisms [81]. Therefore, DLL3 is discussed separately from DLL1 and is not represented by the canonical signal-sending ligand in Figure 2. DLL4 is also not treated as equivalent to DLL1 in this review; because the cited segmentation clock literature does not establish a comparable PSM coupling role, DLL4 is not included in the mechanistic schematic or used to infer clock-specific function.

Zebrafish deltaC and deltaD constitute a separate, species-specific system and should not be collapsed into the mammalian DLL nomenclature. DeltaD and deltaC have distinct contributions to clock dynamics: deltaD has been associated with priming or initiation of her1/her7 activity, whereas deltaC is particularly important for maintaining synchrony between neighboring cells [27]. Their spatial distributions also differ, with deltaD enriched more posteriorly and deltaC expressed broadly across the PSM [28,29]. Moreover, RNA processing can directly alter ligand function: a deltaC splice variant retaining a 90-base-pair intronic sequence produces a C-terminally truncated protein with reduced signaling activity and impaired segmentation [28]. These observations support ligand-specific rather than generic ‘dll’ annotation.

The transcriptional and dosage regulation of zebrafish deltaC and deltaD further illustrates why individual ligands must be considered separately. Altering deltaD expression can disrupt synchrony, and experimental studies indicate that DeltaC and DeltaD differ in their capacities to promote phase coupling [25,29,32,33]. DeltaC can support efficient coupling and partially compensate for loss of deltaD in some settings, whereas deltaD alone is insufficient to reproduce all deltaC-dependent synchronization functions [32]. Regulatory sequences controlling deltaD expression are also incompletely conserved across species [30,31]. Thus, observations from zebrafish deltaC/deltaD should not be directly generalized to mammalian DLL1, DLL3, or DLL4 without ligand- and species-specific evidence.

Beyond classical coupled-oscillator models, recent experimental findings suggest that segmentation clock synchronization involves highly nonlinear interactions between cellular oscillators. Using mouse PSM cells carrying the LuVeLu Notch reporter, Ho et al. demonstrated that synchronization between distinct oscillator populations does not occur through gradual convergence towards an intermediate phase, as predicted by traditional Kuramoto-type models [103,104]. Instead, one oscillator population adopts the phase of another in an asymmetric “winner-takes-all” process. In these experiments, one population maintained its original oscillatory rhythm, while the other shifted its phase to match the dominant oscillator [103]. Randomization assay for low input (RAFL) analysis, which amplifies scarce amount of DNA and distinguishes it from background noise, suggested that the population oscillating ahead in phase becomes the dominant “winner,” while the lagging population undergoes a phase adjustment of approximately π to achieve synchronization [103]. These findings reveal that the segmentation clock employs dynamic and directional communication mechanisms capable of rapidly restoring synchrony following perturbation.

The recovery of synchronization after Notch disruption further demonstrates the complexity of tissue-level clock regulation. Following DAPT washout in zebrafish embryos, regions containing both abnormal and restored somite patterns emerge before complete recovery of segmentation [24]. Computational modelling suggests that these transitional regions arise from transient phase vortices generated during resynchronization, in which local oscillator interactions produce large-scale patterning outcomes [24]. Thus, synchronization is not simply a return to a previous state but involves dynamic reorganization of cellular phase relationships across the tissue. In addition to biochemical communication, physical properties of cells may contribute to synchronization. Recent studies have shown that membrane potential can influence segmentation timing, with increased depolarization accelerating oscillatory periodicity and altering somite growth in chick embryos [105]. These findings suggest that electrical and biochemical signals may converge to regulate the timing and coordination of oscillatory programs.

## 5. Evidence Gaps and Testable Hypotheses: RNA-Mediated Post-Transcriptional Control and Emerging Epitranscriptomic Mechanisms

The mechanisms discussed above primarily regulate the clock at the level of signaling activity, protein turnover, and cellular physiology. These processes interact closely with, but are mechanistically distinct from, RNA-mediated post-transcriptional regulation. We therefore consider RNA stability, processing, translational control, and emerging epitranscriptomic mechanisms separately in this section. Together, these layers determine how oscillatory transcription is converted into appropriately timed protein output and how molecular noise is buffered, while preserving a clear distinction between direct RNA regulation and downstream protein- or signaling-level modulation.

### 5.1. Established Post-Transcriptional Control: Evidence Gaps and Testable Mechanisms

Direct evidence supports a central role for RNA and protein turnover in setting segmentation clock timing, but the contribution of individual post-transcriptional processes remains incompletely resolved. Live imaging of endogenous her1 transcription in zebrafish has shown that single PSM cells generate highly stochastic transcriptional bursts [21]. This observation establishes the noise that the clock must buffer, but does not itself demonstrate a post-transcriptional mechanism. A key unresolved question is therefore how RNA processing, transcript decay, translation, and protein turnover transform variable transcriptional inputs into robust, tissue-level oscillations. The cumulative delays introduced by RNA synthesis, pre-mRNA processing, nuclear export, translation, and protein maturation are predicted to influence oscillator period and stability [47,106], but the quantitative contribution of each step has not been directly defined in the PSM.

One testable hypothesis is that transcript half-life and RNA-processing delay are active determinants of oscillation period rather than passive consequences of gene expression. This could be addressed by measuring the half-lives of core clock transcripts across defined PSM states and oscillatory phases, using metabolic RNA labeling or transcriptional shutoff approaches together with live RNA reporters. Perturbations that selectively prolong or shorten transcript stability could then be assessed for effects on oscillation period, amplitude, phase coherence, and somite boundary formation. Such experiments would directly distinguish causal RNA-kinetic control from correlations between transcript abundance and developmental state.

Translation represents the point at which oscillatory RNA information is converted into protein output and is therefore directly relevant to an RNA–protein kinetic framework. However, evidence for ribosome heterogeneity in the segmentation clock remains indirect. RPL38 selectively regulates translation of specific developmental transcripts without necessarily changing their mRNA abundance, and RPL38 deficiency causes axial skeletal patterning defects [66]. RPL10A is likewise required for normal mesoderm and PSM development, with disruption producing premature differentiation and irregular somite formation [67]. Neither factor has been shown to directly regulate segmentation clock oscillations. We therefore use these studies only to support the broader principle that transcript abundance and protein output can be uncoupled by selective translation, not to claim that RPL38 or RPL10A are core clock components. Phase-resolved ribosome profiling or polysome analysis, coupled to live clock reporters, will be required to establish whether ribosome composition or translational selectivity directly alters oscillator dynamics.

### 5.2. Emerging Epitranscriptomic Mechanisms: Hypothesis-Driven Future Directions

Direct evidence for epitranscriptomic control of the vertebrate segmentation clock remains limited. Nevertheless, several observations from broader RNA biology provide mechanistic reasons to test these pathways in PSM oscillations. N6-methyladenosine (m6A) can alter transcript stability, translation, and cell-state transitions; pseudouridylation can influence RNA structure and translational behavior; RNA editing can change transcript sequence or regulatory interactions; and tRNA modifications can reshape decoding efficiency and codon-dependent translation. These effects establish biological plausibility, but they do not demonstrate that the same mechanisms regulate segmentation clock timing. Under the evidence hierarchy used in this review, such findings constitute Level III inference when demonstrated in another biological system and Level IV hypotheses when extrapolated specifically to the segmentation clock.

The tissue-specific tRNA modification study by Ando et al. [68] illustrates this distinction. The study provides a Level III general principle that tRNA modification states can vary by tissue and associate with codon-biased translational output, but it does not directly demonstrate regulation of the PSM or segmentation clock. Its relevance is therefore hypothesis-generating: developmental tissues might use dynamic tRNA modification states to tune translation of codon-biased clock or boundary-specification proteins. Testing this possibility will require direct measurement of tRNA modification states across segmentation phases, together with codon-usage analysis and translational readouts in PSM or somitoid models.

Several explicit and falsifiable models follow from this indirect evidence. First, oscillatory transcripts may acquire phase-dependent RNA modifications that change transcript half-life and thereby alter feedback delay. Second, RNA modifications may change translational efficiency without substantially altering transcript abundance, producing phase-specific differences between RNA and protein output. Third, changes in tRNA modification could selectively alter translation of codon-biased clock- or boundary-specification genes. Fourth, RNA editing could modify coding sequence or regulatory motifs in a manner that changes the timing or amplitude of clock-associated protein production. These possibilities are mechanistically distinct and can therefore be tested separately rather than being grouped under a generic epitranscriptomic mechanism.

Direct testing should combine modification-specific RNA profiling at defined clock phases with transcript half-life measurements, ribosome profiling, quantitative proteomics, live RNA imaging, and CRISPR-based perturbation of candidate RNA-modifying enzymes in experimentally tractable PSM, segmentoid, or somitoid systems. A proposed mechanism should be upgraded from hypothesis to direct clock evidence only when perturbation of the relevant modifier produces a reproducible change in an RNA-level feature together with a defined clock phenotype, such as altered period, amplitude, synchronization, wave propagation, or boundary formation. Rescue with catalytically active versus inactive modifier constructs would further help us to distinguish modification-dependent effects from non-catalytic functions. This framework turns the current lack of direct evidence into a set of discriminating experiments rather than treating epitranscriptomics as an established causal layer.

## 6. Pattern and Boundary Formation: From RNA Waves to Stable Somites

The ultimate output of segmentation clock activity is the periodic generation of morphologically distinct somites, which establish the segmented architecture of the vertebrate axial skeleton and associated tissues. Although oscillatory gene expression has long been recognized as the temporal engine driving somitogenesis, a fundamental question remains: How are transient waves of RNA expression converted into stable anatomical boundaries? The classical clock-and-wavefront model proposed that oscillations provide temporal information, while a maturation wavefront arrests clock activity at a specific position along the anterior–posterior (AP) axis, thereby determining future somite boundaries [2]. However, accumulating evidence from vertebrate embryos and stem-cell-derived models suggests that boundary formation is not simply a consequence of oscillator arrest. Instead, stable segmentation emerges through the integration of oscillatory transcriptional programs, RNA dynamics, signaling gradients, lineage specification networks, and tissue-level mechanical processes.

Human segmentoid and somitoid models have provided important insights into how dynamic RNA programs transition into differentiated somite identities. These in vitro systems recapitulate neuromesodermal progenitor, posterior PSM, anterior PSM, and somite-like states characterized by sequential expression of human TBXT, HES7, TBX6, MESP2, PAX3, UNCX, and TBX18 [12,16,107]. In human models, MESP2 activation occurs shortly after cessation of HES7 oscillations [12]. The subsequent mechanistic studies discussed here come primarily from zebrafish and should not be read as direct demonstrations of the human pathway. In zebrafish, peer-reviewed single-cell and live-imaging analyses show a finite number of her1 oscillatory cycles before slowing and arrest during differentiation [22], whereas a preprint uses high-resolution imaging to link dynamic tbx6 waves to future boundary positions [64]. We therefore treat the latter as provisional Level I clock-to-boundary evidence and do not generalize it to human somitogenesis without independent validation.

Tbx6 represents a critical molecular intermediate linking oscillatory output to somite boundary formation. Peer-reviewed work establishes roles for Tbx6 and Ripply family proteins in boundary specification and Tbx6 turnover in vertebrate models [34,36,37,38,39,108,109,110,111]. A recent zebrafish preprint further reports that clock-driven waves of tbx6 expression prefigure future somite boundaries [64]. Because that specific dynamic-wave observation remains a preprint, it is treated as provisional even though the underlying Tbx6/Ripply boundary-conversion framework is supported by peer-reviewed studies. This distinction prevents a recent high-resolution observation from being assigned the same publication certainty as the established developmental mechanism.

The significance of controlled Tbx6/tbx6 turnover is illustrated primarily by zebrafish and Xenopus studies. In zebrafish, ripply1 and ripply2 link segmentation clock activity to boundary formation by promoting tbx6 degradation and restricting tbx6-dependent transcription [34,110]. In Xenopus, related Ripply/Bowline factors interact with Tbx6 and Groucho-family co-repressors, supporting conservation of a boundary-conversion module without implying identity of the upstream clock circuitry [36,37,38,39,109]. Within zebrafish anterior PSM, her1/her7-dependent regulation of ripply generates dynamic expression domains that move with clock waves. Two-color FISH distinguishing nascent and mature zebrafish her1 transcripts further indicates that clock activity can extend anterior to the future boundary [34]. These observations define a zebrafish mechanism of tbx6-state transition; extrapolation to human TBX6/MESP2 timing requires direct validation in human models.

In zebrafish, peer-reviewed studies support coupling between ripply expression, ERK signaling, and tbx6 clearance during somite boundary formation [34,111]. ERK inhibition expands nascent ripply1/2 expression posteriorly, whereas increased ERK activity suppresses ripply expression despite elevated tbx6 levels. Mathematical modeling further supports a relationship between oscillatory dynamics and Tbx6 clearance [34], but model-derived predictions are interpreted in the context of the accompanying experimental data. By contrast, a reviewed preprint examining ripply1 and gsc in zebrafish prechordal plate/anterior endoderm [69] addresses a distinct developmental context and is classified here as Level III evidence; it is not used to establish a segmentation clock mechanism.

Once molecular boundaries are established, additional transcriptional and mechanical programs transform these prepatterns into mature somites. In human somitoids, *MESP2* activation is followed by expression of anterior somite markers, including *PAX3* and *TCF15*, accompanied by progressive establishment of AP polarity [12]. Similar roles have been demonstrated for zebrafish *mespb* and mouse *Mesp2*, which are essential for boundary formation and rostrocaudal identity [112]. Disruption of cell polarity regulators such as Tiam1 impairs anterior–posterior patterning, demonstrating that molecular specification must be integrated with cellular organization. Somite maturation further requires epithelialization and tissue architecture. Human somitoids develop characteristic epithelial features, including apical enrichment of F-actin, N-cadherin localization, laminin-rich basement membranes, and ZO-1-positive luminal structures, closely resembling embryonic somites in vivo [12,16]. Thus, transcriptional patterning alone is insufficient; molecular boundaries must be reinforced through coordinated changes in cell adhesion, polarity, and tissue mechanics.

Interestingly, somite morphology exhibits additional mechanisms of robustness beyond transcriptional patterning. High-resolution imaging in zebrafish has shown that newly formed somites display considerable variability in initial AP length, reflecting imperfect precision in mesp-based prepatterning [112,113]. However, this variability decreases over time through compensatory changes in mediolateral dimensions that restore bilateral symmetry and reduce developmental noise. These corrections occur without substantial contributions from altered proliferation or tissue growth, suggesting that mechanical rearrangement and cell shape changes provide downstream buffering mechanisms [113,114]. Importantly, these corrective processes remain active even in segmentation clock mutants, indicating that mechanical refinement operates after molecular patterning has been established. Collectively, current evidence supports a revised model of somite boundary formation in which oscillatory RNA dynamics provide temporal information but do not directly determine anatomical boundaries. Instead, oscillatory programs are interpreted through TBX6 and RIPPLY-mediated regulatory switches, integrated with FGF/ERK signaling gradients, and stabilized through MESP-dependent lineage specification. Subsequent epithelialization and mechanical refinement converts these molecular states into robust anatomical structures.

## 7. Stopping the Clock: PSM Depletion, Loss of Oscillatory Competence, and Transition to Differentiation

Termination of the segmentation clock is a distinct developmental problem from the arrest of a single oscillatory cycle. At least three related processes should be separated conceptually: cessation of oscillatory transcription in individual PSM cells as they approach differentiation; progressive loss of the tissue environment and signaling competence required to sustain coordinated oscillations; and final termination of somitogenesis as posterior axial progenitor input declines and the PSM is depleted. These events are temporally coupled but need not be driven by one molecular ‘off switch’.

One possibility is that clock arrest reflects a progressive timer-like loss of oscillatory competence. In zebrafish, posterior PSM cells can undergo more oscillatory cycles than anterior cells, and the number of remaining cycles decreases as the cells progress toward differentiation [22,93]. This behavior is consistent with a countdown-like process, but it does not by itself establish whether the timer is cell autonomous. FGF signaling can extend oscillatory competence, whereas its attenuation accelerates differentiation [89,94], suggesting that intrinsic timing and changing extracellular signals may act together. A key unresolved question is therefore whether the final oscillatory cycle is determined primarily by an internal maturation program, by progressive exposure to a changing signaling environment, or by an interaction between the two.

At the tissue level, late somitogenesis must also be understood in the context of continued axial growth and eventual PSM depletion. Comparative morphometric studies across chicken, mouse, zebrafish, and corn snake show that somite scaling changes during late axis elongation, although the strongest perturbation evidence discussed here derives from zebrafish [23,41]. In zebrafish PSM explants, tissue context and posteriorly supplied signals influence somite size, and tailbud removal shifts the determination front posteriorly and produces smaller somites [23]. These findings support the broader principle that continued segmentation depends on the maintenance of posterior progenitor input and signaling context, but they do not demonstrate that fgf/wnt decline alone is the molecular trigger that terminates the clock.

Accordingly, signaling decline should be distinguished from direct oscillator shutdown. In zebrafish PSM, fgf8 signaling is spatially constrained by ligand distribution, receptor availability, and receptor-mediated endocytosis near the anterior PSM boundary, and phosphorylated ERK measurements suggest that cells can respond to relative changes in fgf signaling [23]. However, the same study did not establish an identical scaling rule for Wnt signaling, and these quantitative relationships should not be generalized directly to mouse, chick, or human PSM without species-specific validation [23,115]. The available data therefore support a role for FGF/ERK and WNT pathways in maintaining PSM identity, positional information, and oscillatory competence, while the mechanism that determines the final clock cycle remains incompletely resolved.

Late-axis termination may also involve developmental programs that operate upstream of, or in parallel with, the core oscillator. A recent preprint in the Japanese eel reports prolonged axis segmentation associated with delayed Hox13 activation and sustained Oct4 expression [42]. Because this work addresses a species-specific late-axis phenotype rather than a direct measurement of core clock shutdown, it is treated here as provisional Level II evidence. Such comparative systems nevertheless provide a useful framework for testing whether the end of somitogenesis is governed chiefly by posterior progenitor exhaustion, changes in axial identity, altered growth dynamics, or combinations of these processes.

From the perspective of RNA–protein dynamics, several mechanisms could plausibly accompany loss of oscillatory competence, including altered transcriptional responsiveness, changes in transcript processing or half-life, reduced translation of clock components, altered protein degradation, and declining Notch-coupling competence. Direct evidence that a dedicated RNA-decay program terminates vertebrate somitogenesis is currently limited; these mechanisms should therefore be treated as testable hypotheses rather than established shutdown pathways. Longitudinal measurements across the final oscillatory cycles will be required to determine whether changes in RNA and protein kinetics precede clock arrest or instead occur as consequences of differentiation.

A useful working model is therefore that clock termination is a systems-level transition rather than a single switch: posterior progenitor supply diminishes, the PSM contracts, signaling conditions change, and cells progressively lose the capacity to synchronize another coordinated oscillatory cycle. This model predicts that termination should be preceded by an ordered sequence of changes in cell state, signaling responsiveness, RNA kinetics, and protein dynamics. Testing this prediction will require simultaneous live measurements of nascent transcription, RNA turnover, protein abundance, signaling activity, and cell fate during the last several segmentation cycles, coupled with temporally controlled perturbation of candidate pathways. The hypothesis is carried forward explicitly in Section 9, where clock termination is framed as a coordinated kinetic transition that can be tested in vertebrate embryos and human stem-cell-derived somitogenesis systems. The species-qualified RNA and RNA-associated modules discussed throughout this review are summarized in Table 3.

## 8. Human Genetics and Disease: Clinically Curated Evidence for Vertebral Segmentation Disorders

Human vertebral segmentation disorders arise from pathogenic variation acting at distinct molecular levels. Some variants directly alter RNA splicing or transcript processing, whereas others primarily disrupt protein stability, enzymatic activity, transcriptional repression, or Notch-pathway signaling. The clinical genetics framework therefore separates demonstrated RNA consequences from protein- or signaling-level consequences and avoids classifying all disease mechanisms as RNA defects. To maintain clinical-genetic rigor and relevance to the segmentation clock, the discussion below focuses on the established autosomal-recessive spondylocostal dysostosis/vertebral-segmentation genes HES7, LFNG, DLL3, MESP2, and RIPPLY2 (Table 4) [116,117,119,120,121,122,123,124,125,126].

Autosomal-recessive spondylocostal dysostosis (SCDO) is characterized by multiple vertebral segmentation defects, often with rib abnormalities, scoliosis, and short-trunk stature. GeneReviews recognizes biallelic disease-causing variants in DLL3, HES7, LFNG, MESP2, RIPPLY2, and additional genes as causes of SCDO; in this review, Table 4 is deliberately restricted to representative variants in HES7, LFNG, DLL3, MESP2, and RIPPLY2, for which the reference transcript, HGVS nomenclature, inheritance, and molecular evidence can be stated explicitly [127]. This focused approach avoids grouping genes with different mechanisms or levels of gene–disease evidence under generic family labels. Within the evidence hierarchy used here, human gene–disease associations are classified as Level II with respect to segmentation clock causality unless oscillatory dynamics have been directly measured in an experimental model carrying the relevant perturbation; clinical pathogenicity and clock-mechanism evidence are therefore reported as distinct dimensions.

The molecular evidence illustrates why mechanistic annotation must be variant-specific. For HES7, the homozygous splice-donor variant c.226+1G>A was shown by minigene analysis to cause intron 3 retention, providing direct evidence of an RNA-processing defect [122]. By contrast, the LFNG p.Phe188Leu variant was shown to impair protein localization, glycosyltransferase activity, and modulation of Notch signaling, without a demonstrated primary RNA-processing defect [124]. DLL3 loss-of-function variants provide a well-established genetic link between the somitogenesis network and SCDO1, whereas MESP2 and RIPPLY2 variants disrupt downstream interpretation of segmentation cues through protein-level transcriptional functions [116,117,125]. These examples demonstrate that similar vertebral phenotypes can arise through mechanistically distinct defects.

Table 4 is intended as a representative clinically curated evidence table rather than an exhaustive variant catalogue. Entries were retained only when the human gene symbol and transcript could be specified, inheritance was interpretable, and RNA-level consequences could be distinguished from protein-/signaling-level consequences. Molecular consequences are now explicitly qualified according to the evidence reported in the cited studies. “Experimentally demonstrated” indicates direct RNA-, protein-, or signaling-level validation in the original or cited functional study. “Predicted/inferred” indicates a consequence inferred from variant type, sequence context, or established molecular principles but not directly demonstrated in the cited study. “Not directly demonstrated” indicates that the relevant molecular level was not experimentally tested. In particular, splice disruption and nonsense-mediated decay are not treated as experimentally established unless supported by patient RNA analysis, minigene testing, RT-PCR, sequencing of an aberrant transcript, or an equivalent transcript-level assay. Clinical pathogenicity, molecular mechanism, and direct segmentation clock evidence are therefore presented as distinct dimensions rather than as equivalent levels of evidence.

Human stem-cell-derived somitogenesis models, including segmentoids, somitoids, and axioloids, now provide an opportunity to test clinically observed variants under controlled developmental conditions [12,16,107]. Patient-derived or engineered variants can be evaluated using quantitative readouts such as HES7 oscillation period, LFNG phase dynamics, MESP2 stripe formation, transcript half-life, protein localization or stability, Notch-pathway output, and epithelial somite architecture. These models are particularly valuable for variants whose clinical association is established but whose direct RNA or protein consequence remains unresolved.

Collectively, the human genetic evidence supports a clinically heterogeneous view of segmentation clock disease. HES7, LFNG, DLL3, MESP2, and RIPPLY2 converge on vertebral segmentation but do so through distinct molecular mechanisms, including altered RNA splicing, loss of enzymatic or signaling activity, and impaired transcriptional repression. Distinguishing these mechanisms prevents over-attribution of congenital vertebral disease to RNA dysregulation and provides a more rigorous basis for connecting human genetics with the dynamic RNA–protein biology of somitogenesis.

## 9. Future Perspectives: Segmentation Clock RNA Biology as a Framework for Developmental Timing

The most important next step is not simply to apply newer technologies to the segmentation clock, but to use them to discriminate among competing mechanistic models of how RNA processing, translation, intercellular coupling, metabolism, and RNA modification contribute to clock timing and robustness. We therefore propose five specific, testable hypotheses that follow directly from the evidence gaps identified in this review.

**Hypothesis** **1.** *RNA-processing and turnover kinetics contribute causally to segmentation clock period. Species- and model-specific differences in oscillation period may arise, at least in part, from differences in pre-mRNA processing, transcript maturation, nuclear export, and RNA degradation. This hypothesis can be tested by measuring processing delays and transcript half-lives for core clock genes across vertebrate species and human stem-cell-derived PSM models, followed by inducible perturbation of splicing or RNA-decay machinery. A causal contribution would be supported if experimentally prolonging or shortening these kinetic delays produces predictable changes in oscillation period, phase, or robustness.*

**Hypothesis** **2.** *Notch signaling primarily stabilizes and synchronizes oscillators after their initiation. This hypothesis can be tested using live single-cell reporters of nascent transcription combined with temporally controlled Notch inhibition or activation before and after oscillator onset. If initiation and synchronization are mechanistically separable, early oscillatory transcription should emerge before full Notch-dependent coupling, whereas perturbation after onset should preferentially alter phase coherence, wave propagation, and tissue-level synchronization rather than abolish the first oscillatory cycle.*

**Hypothesis** **3.** *Translation, protein turnover, and metabolic state actively tune oscillator period and robustness rather than acting only as downstream consequences of transcription. Phase-resolved ribosome profiling, quantitative proteomics, live protein reporters, and controlled metabolic perturbations can be combined to determine whether changes in translational efficiency, protein half-life, glycolytic flux, or oxygen-sensitive signaling alter oscillation period directly or primarily affect amplitude, recovery from perturbation, and developmental robustness. These experiments would distinguish mechanisms that set clock speed from those that buffer the oscillator against physiological noise.*

**Hypothesis** **4.** *Epitranscriptomic modifications regulate phase-specific RNA fate in the segmentation clock. One testable possibility is that modifications such as m6A, pseudouridylation, RNA editing, or tRNA modification alter the stability, localization, decoding, or translational efficiency of oscillatory transcripts in a phase-dependent manner. This can be addressed by modification-specific RNA profiling at defined clock phases, coupled with transcript half-life measurements, ribosome profiling, and CRISPR-based perturbation of candidate RNA-modifying enzymes. A causal role would require both a demonstrable RNA-level change and a reproducible clock phenotype, such as altered period, amplitude, synchronization, or boundary formation.*

**Hypothesis** **5.** *Clock termination reflects a coordinated transition in molecular kinetics rather than a single stop signal. Longitudinal live imaging of RNA and protein reporters during PSM maturation, combined with perturbation of candidate differentiation and termination pathways, can test whether loss of oscillatory competence is preceded by a reproducible sequence of changes in transcript turnover, protein stability, signaling responsiveness, or translational state. Human segmentoid, somitoid, and axioloid systems provide an especially useful platform for testing these hypotheses and for evaluating patient-associated variants using quantitative clock readouts, while comparison with mouse and zebrafish models can distinguish conserved from species-specific mechanisms. Ethical oversight of human stem cell-based embryo models should not be described by applying the conventional human embryo 14-day framework indiscriminately. Under the current ISSCR Guidelines, human embryos and stem cell-based embryo models (SCBEMs) are treated as distinct research categories. Research involving more complex, spatially organized SCBEMs requires specialized scientific and ethical oversight proportionate to model complexity, a clear scientific rationale for use of the model, and a defined culture endpoint linked to the developmental milestone under investigation. Investigators should also comply with applicable local and national regulations and engage the appropriate oversight body during protocol design. Accordingly, gastruloids, somitoids, segmentoids, axioloids, and related SCBEMs should not automatically be assigned a universal 14-day limit. By contrast, the ISSCR’s discussion of culture beyond 14 days specifically concerns research involving human embryos and requires jurisdictional permissibility, compelling scientific justification, and specialized oversight; this framework should not be generalized without qualification to SCBEMs.*

In conclusion, the central theme of this review is the temporal control of gene-expression output across the RNA–protein axis. RNA synthesis, processing, stability, degradation, and translation constitute the primary RNA-centered mechanisms considered here. Protein turnover, metabolism, and signaling are included only where they measurably constrain the timing, robustness, or interpretation of those RNA-derived oscillations. This framing avoids treating all developmental regulators as components of RNA biology while preserving the key insight that segmentation clock behavior emerges from coordinated kinetics across molecular layers. Epitranscriptomic regulation remains an emerging hypothesis-driven frontier whose direct contribution to PSM oscillations has yet to be established.

## Figures and Tables

**Table 1:** Species-specific segmentation clock genes and experimental evidence.

Organism/Model	Principal Genes/Components	Species-Specific Evidence Represented in This Review	Clock Period/Timing	Reference
Human iPSC-derived PSM models (in vitro)	HES7, LFNG, TBX6, MESP2	HES7/LFNG oscillations and somite-like boundary programs in segmentoid/somitoid/axioloid systems; not direct in vivo human embryo evidence.	~4–6 h	[11,12,15,16]
Mouse embryo/PSM explant	Hes7, Lfng, Dll1	Early Lfng waves, Hes7 feedback, and Dll1/Lfng-dependent Notch coupling. These data define a mouse circuit and are not substituted for zebrafish paralogs.	~2–3 h	[17,18,19,20]
Zebrafish embryo/PSM	her1, her7, deltaC, deltaD; tbx6/ripply	Live her1 transcription, her1/her7 feedback, Delta-dependent synchronization, and tbx6/ripply boundary-conversion dynamics.	~30 min	[21,22,23,24,25,26,27,28,29,30,31,32,33,34]
Chicken embryo	c-hairy1, LFNG	Original cyclic c-hairy1 expression and avian segmentation timing; avian nomenclature retained as reported.	~90 min	[5,14,20]
Xenopus embryo	tbx6, mespb, ripply/bowline	Downstream boundary-conversion and Tbx6/Ripply-Groucho interactions; not used as direct evidence for mouse/human core-clock kinetics.	Not emphasized	[35,36,37,38,39]
Goldfish embryo	lrp6a/Wnt pathway	Wnt-pathway perturbation affects somitogenesis and skeletal patterning; supportive signaling evidence rather than core-clock ortholog evidence.	Not established	[40]
Corn snake embryo	Clock genes not resolved in cited comparison	Comparative somite scaling and high vertebral count; no gene-level equivalence inferred.	~100 min	[14,20,41]
Japanese eel embryo	Hox13, Oct4	Late-axis termination/prolonged segmentation phenotype; preprint evidence, not evidence for mouse or zebrafish core-clock orthology.	Not established	[42]

Gene nomenclature follows the organism used in the cited experiment: human genes are shown in HGNC-style uppercase symbols, mouse genes with initial capitalization, and zebrafish genes in lowercase; chick and other vertebrate nomenclature follow the original cited literature. Her, hairy and enhancer of split-related bHLH transcriptional repressor; Hes, hairy and enhancer of split 1; DLL1, Delta-like canonical Notch ligand 1; LFNG, lunatic fringe; MESP2, Mesoderm posterior bHLH transcription factor2; TBX6, t-box 6; PSM, presomitic mesoderm; iPSC, induced pluripotent stem cell.

**Table 2:** Evidence hierarchy for representative mechanisms discussed in this review.

Molecular Mechanism	Molecule/Gene	Species/Model	Experimental Approach	Measured Clock or Developmental Phenotype	Evidence Level	Publication Status/Reference
Transcriptional bursting/oscillator noise	her1	Zebrafish embryo PSM	Endogenous MS2-MCP reporter; light-sheet live imaging	Bursting kinetics and oscillatory transcription measured in single cells	I: Direct clock evidence	Peer-reviewed; Cell Systems 2026 [21]
Early oscillator onset	Lfng; Hes7	Mouse embryo PSM	DAPT/Rbpj perturbation; Hes7 loss; spatiotemporal expression analysis	Early Lfng waves persist before full canonical Notch coupling	I: Direct clock evidence	Peer-reviewed [18]
Notch-mediated coupling/synchronization	Dll1; Lfng; Notch	Mouse PSM	Genetic perturbation and oscillatory reporter analyses	Intercellular phase coupling/synchronization	I: Direct clock evidence	Peer-reviewed [19,24,25,26]
Species-specific Delta coupling	deltaC; deltaD; her1/her7	Zebrafish embryo PSM	Ligand perturbation, notch inhibition, live/expression analyses	Desynchronization and altered her1/her7 oscillations	I: Direct clock evidence	Peer-reviewed [24,25,26,27,28,29,30,31,32,33]
NICD proteolytic timing	FBXW7; NOTCH1	Human iPSC-derived PSM	FBXW7 inhibition/NOTCH1 PEST-domain perturbation; oscillatory reporters	Altered HES7 wave propagation and clock dynamics	I: Direct clock evidence	Peer-reviewed [59,60]
Glycolytic control of clock speed	Glycolytic metabolites; Wnt/Notch	Mouse PSM explants	Metabolic supplementation/perturbation with live signaling readouts	Changed oscillation speed and somite length	I: Direct clock evidence	Peer-reviewed [61]
Oxygen/metabolic homeostasis	Hif1a; Hes7; Lfng	Mouse PSM conditional knockout	Genetic deletion, oxygen rescue, expression/segmentation analyses	Abnormal clock oscillations; rescue by hyperoxia	I: Direct clock evidence	Preprint; provisional [62]
Cell-cycle/clock coupling	Hes7/signaling oscillations; cell-cycle regulators	Mouse embryo tails/PSM	Long-term single-cell tracking; microfluidic entrainment; proliferation blockade	Clock-cell-cycle coupling and altered somite scaling	I: Direct clock evidence	Preprint; provisional [63]
Clock-to-boundary conversion	tbx6/ripply/mesp	Zebrafish embryo	Live imaging and perturbation of dynamic Tbx6/Ripply states	Oscillatory output linked to boundary specification	I: Direct clock-to-boundary evidence	Preprint for Ref. [64]; complementary peer-reviewed evidence [34,64]
Spatiotemporal protein gradients	R-Spondin/LGR; Wnt pathway	Mouse embryo tails/gastruloids	Microfluidic synchronization, proteomics/RNA-seq, perturbation	Wnt oscillation amplitude and somite formation altered	I–II: Direct signaling dynamics/somitogenesis	Preprint; provisional [65]
Selective ribosome-mediated translation	RPL38; RPL10A	Mouse axial tissues/stem-cell mesoderm models	Genetic perturbation; translational and developmental phenotyping	Axial/mesodermal patterning defects; clock oscillations not directly measured	II: Somitogenesis/axial development, not direct clock evidence	Peer-reviewed [66,67]
Human congenital segmentation disease	HES7; LFNG; DLL3; MESP2; RIPPLY2	Human clinical genetics	Variant segregation, clinical phenotyping, selected functional assays	Vertebral segmentation phenotype; patient clock oscillations generally not measured	II: Human axial/segmentation disease evidence	Peer-reviewed/curated sources; see Table 4
Tissue-specific tRNA modification	tRNA modifications	Non-PSM tissues	Modification profiling and translational analyses	No segmentation clock phenotype measured	III: Inferred from another biological context	Peer-reviewed [68]
Epitranscriptomic control	m6A, pseudouridylation, RNA editing	Other developmental/cellular systems	Modification-specific profiling in non-PSM contexts	No direct PSM clock phenotype established	III–IV: Inferred/hypothesis requiring validation	See Section 5 [56,57]
Late-axis termination/prolonged segmentation	Hox13; Oct4	Japanese eel embryo	Comparative developmental profiling	Prolonged axis segmentation; core clock oscillations not established	II: Axial development, not direct clock evidence	Preprint; provisional [42]
Ripply function outside PSM segmentation	ripply1; gsc	Zebrafish prechordal plate/anterior endoderm	Single-cell multi-omics, live imaging, perturbation	Cell-fate effects outside the segmentation clock	III: Non-segmentation developmental context	eLife Reviewed Preprint; not clock evidence [69]

DLL1, Delta-like canonical Notch ligand 1; FBXW7, F-box and WD repeat domain-containing protein 7; Her, hairy and enhancer of split-related bHLH transcriptional repressor; Hes, hairy and enhancer of split 1; iPSC, induced pluripotent stem cell; LFNG, lunatic fringe; MESP2, Mesoderm posterior bHLH transcription factor2; MCP, MS2 Coat Protein; NICD, Notch intracellular domain; PSM, presomitic mesoderm; RPL, Ribosomal Protein L; TBX6, t-box 6.

**Table 3:** Species-qualified RNA and RNA-associated modules discussed in the segmentation clock.

Species-Specific Module	RNA/Protein Function	Developmental Role	Impact of Mis-Regulation	Reference
Mouse Lfng	Early Lfng pulses can precede full canonical Notch coupling; later LFNG modulates Dll1/Dll3-dependent signaling.	Mouse PSM coupling, stabilization, and synchronization after oscillator onset.	Mistiming disrupts phase coherence and somite formation.	[18,19,81]
Mouse Hes7	Delayed negative feedback with rapid RNA/protein turnover.	Principal mouse intracellular oscillator controlling periodicity.	Altered kinetics disrupt periodicity and segmentation.	[7,17,84,89]
Zebrafish her1/her7	Oscillatory transcription, feedback repression, and live-observed transcriptional bursting.	Principal zebrafish oscillator and phase dynamics.	Perturbed transcription/coupling causes desynchronization and abnormal boundaries.	[21,22,86,87,88]
Mouse Dll1	Canonical trans-activating Notch ligand; signal-sending activity modulated by Lfng.	Mouse intercellular coupling and synchronization.	Loss impairs Notch coupling and segmentation.	[19]
Mammalian Dll3/human DLL3	Atypical intracellular/cis Notch regulator; cooperates with LFNG to tune DLL1/NOTCH1 signaling.	Modulates mammalian Notch dynamics; not a canonical trans ligand.	Biallelic human DLL3 loss-of-function causes SCDO1.	[81,116,117]
Zebrafish deltaC/deltaD	Distinct ligands with different timing, distribution, and RNA-processing properties.	Species-specific priming/coupling; deltaC is particularly important for phase synchrony.	Loss, overexpression, or abnormal splicing causes ligand-specific segmentation defects.	[25,27,28,29,32,33]
Zebrafish tbx6/ripply; related Xenopus Ripply/Bowline	Clock-output conversion coupled to ripply-mediated tbx6 degradation.	Converts zebrafish oscillatory output into stable boundary states; related downstream mechanism supported in Xenopus.	Failed state transition disrupts boundary precision.	[34,36,37,38,39,69,107,109,110,111,118]
Zebrafish fgf8/ERK program	Gradient-dependent signaling and transcript programs shape determination-front timing.	Positions zebrafish determination front and scales somite size.	Perturbation shifts boundary position and somite size.	[23,90]
Human/mammalian RPL38 and RPL10A-associated translation	Transcript-selective translation and ribosome heterogeneity.	Controls selected developmental transcripts and axial patterning.	Axial skeletal defects or altered mesodermal progression.	[15,66,67]

Gene symbols and mechanistic statements are tied to the organism in which the cited evidence was obtained. Mouse Hes7/Lfng/Dll1, zebrafish her1/her7/deltaC/deltaD, human HES7/LFNG, and chick c-hairy1/LFNG are not treated as interchangeable components. Human disease genes are shown using HGNC-approved uppercase symbols. ERK, extracellular signal-regulated kinase; Her, hairy and enhancer of split-related bHLH transcriptional repressor; Hes, hairy and enhancer of split 1; DLL1, Delta-like canonical Notch ligand 1; LFNG, lunatic fringe; MESP, Mesoderm posterior bHLH transcription factor; TBX6, t-box 6; PSM, presomitic mesoderm; RPL, Ribosomal Protein L; SCDO, spondylocostal dysostosis.

**Table 4:** Selected human variants in genes associated with spondylocostal dysostosis and vertebral segmentation defects: clinically curated molecular evidence with explicit molecular-consequence evidence status.

**HGNC Gene/Transcript/HGVS**	**Zygosity/Inheritance**	**Variant Classification & Supporting Evidence**	**RNA Consequence/Evidence Status**	**Protein/Signaling Consequence/Evidence Status**	**Phenotype/Gene–Disease Relationship**	**Reference**
HES7 NM_001165967.2 c.226+1G>A p.(Ala76AspfsTer168)	Homozygous; AR	Likely pathogenic in primary study (ACMG/AMP: PVS1_strong + PM2_supporting + PP4); segregation and minigene evidence	Experimentally demonstrated: intron 3 retention in a minigene assay.	Predicted/inferred: frameshift and premature termination based on the aberrant transcript; the downstream protein consequence was not directly demonstrated in the cited study.	SCDO4; established AR HES7–SCDO relationship	[122,127]
LFNG NM_001040167.2 c.564C>A p.(Phe188Leu)	Homozygous; AR	Pathogenic; family segregation plus functional validation; ClinVar pathogenic assertion for SCDO3	Not directly demonstrated: no RNA-processing defect was shown in the cited studies.	Experimentally demonstrated: abnormal localization, loss of glycosyltransferase activity, and impaired Notch modulation.	SCDO3; established AR LFNG–SCDO relationship	[124,127]
DLL3 NM_016941.4 c.535G>T p.(Glu179Ter)	Homozygous; AR	Pathogenic in primary study under ACMG/AMP criteria; segregation in a consanguineous family	Predicted/inferred: nonsense-mediated decay is a plausible consequence of the premature termination codon, but it was not directly demonstrated in patient RNA.	Predicted/inferred: premature truncation is expected to remove C-terminal functional domains and impair DLL3 function; this specific protein consequence was not directly demonstrated in the cited family study.	SCDO1; established AR DLL3–SCDO relationship	[117,127,128]
MESP2 NM_001039958.2 c.500_503dupACCG p.(Gly169ProfsTer199)	Homozygous; AR	Pathogenic in ClinVar; segregation in a consanguineous family with functional follow-up	Not directly demonstrated: no RNA-level consequence was shown in the cited studies.	Experimentally demonstrated: the frameshifted mutant protein showed loss of normal transcriptional activity in functional assays.	SCDO2; established AR MESP2–SCDO relationship	[116,127]
RIPPLY2 NM_001009994.3 c.238A>T p.(Arg80Ter)	Homozygous in some affected individuals; also reported in trans with c.240-4T>G; AR	Condition-specific ClinVar: pathogenic for SCDO6; aggregate variant-level submissions include conflicting classifications	Not directly demonstrated: no transcript-level consequence was shown in the cited studies.	Mixed evidence: loss of the C-terminal Ripply homology domain is inferred from the truncating variant, whereas reduced transcriptional repression was experimentally demonstrated in vitro.	SCDO6/vertebral segmentation defects; established AR RIPPLY2–SCDO relationship	[125,126,127]

ACMG/AMP, American College of Medical Genetics and Genomics and the Association for Molecular Pathology; AR, autosomal recessive; DLL1, Delta-like canonical Notch ligand 1; Hes, hairy and enhancer of split 1; HGVS, Human Genome Variation Society; LFNG, lunatic fringe; LP, likely pathogenic; MESP2, Mesoderm posterior bHLH transcription factor2; NMD, nonsense-mediated decay; PVS, Pathogenic Very Strong; PM2, Moderate Pathogenic Evidence #2; PP4, Supporting Pathogenic Evidence #4, SCDO, spondylocostal dysostosis. Variant classifications are reported at the level supported by the cited primary study and/or condition-specific ClinVar record; absence of a demonstrated RNA consequence is stated explicitly rather than inferred. Gene–disease relationships are summarized using GeneReviews and the cited primary literature [127].

## Data Availability

No new data were created or analyzed in this study. Data sharing is not applicable to this article.
